# APPLaUD: access for patients and participants to individual level uninterpreted genomic data

**DOI:** 10.1186/s40246-018-0139-5

**Published:** 2018-02-17

**Authors:** Adrian Thorogood, Jason Bobe, Barbara Prainsack, Anna Middleton, Erick Scott, Sarah Nelson, Manuel Corpas, Natasha Bonhomme, Laura Lyman Rodriguez, Madeleine Murtagh, Erika Kleiderman, on behalf of the Participant Values Task Team of the Global Alliance for Genomics and Health

**Affiliations:** 1 Centre of Genomics and Policy, Department of Human Genetics, McGill University Faculty of Medicine, Montreal, Quebec H3A 0G1 Canada; 2 Icahn School of Medicine at Mount Sinai, New York, USA; 3 Department of Political Science, University of Vienna, Vienna, Austria; 4 Society and Ethics Research, Connecting Science, Wellcome Genome Campus, Hinxton, UK; 5 Icahn Institute for Genomics & Multiscale Biology, New York, USA; 6 University of Washington, Seattle, USA; 7 Cambridge Precision Medicine, Cambridge, UK; 8 Genetic Alliance, Washington DC, USA; 9 National Human Genome Research Institute, National Institutes of Health, Bethesda, USA; 10 Newcastle University, Newcastle upon Tyne, UK; 11 https://www.ga4gh.org; 12 Department of Global Health & Social Medicine, King’s College London, London, UK; 13 Faculty of Education, University of Cambridge, Cambridge, UK

**Keywords:** Ethics, Law, Genomic data, Individual access, Whole genome sequencing, Direct-to-consumer, Privacy, Raw genomic data, Patient engagement, Citizen science

## Abstract

**Background:**

There is a growing support for the stance that patients and research participants should have better and easier access to their raw (uninterpreted) genomic sequence data in both clinical and research contexts.

**Main body:**

We review legal frameworks and literature on the benefits, risks, and practical barriers of providing individuals access to their data. We also survey genomic sequencing initiatives that provide or plan to provide individual access. Many patients and research participants expect to be able to access their health and genomic data. Individuals have a legal right to access their genomic data in some countries and contexts. Moreover, increasing numbers of participatory research projects, direct-to-consumer genetic testing companies, and now major national sequencing initiatives grant individuals access to their genomic sequence data upon request.

**Conclusion:**

Drawing on current practice and regulatory analysis, we outline legal, ethical, and practical guidance for genomic sequencing initiatives seeking to offer interested patients and participants access to their raw genomic data.

## Background

The quantity of genomic data generated about individual patients, research participants, and consumers is rapidly increasing. The Global Alliance for Genomics and Health (GA4GH), an international public-private consortium, develops technical standards and frames policy to facilitate the sharing of health and genomic data between health care, research, and individuals. Analyzing and sharing these data leads to novel health insights and opportunities [1], but it raises ethical questions about the flow of data back to individuals. Debate has centered on what types of individual findings should be reported from testing or research [2] and has tended to focus on the clinical validity and actionability of results, and whether or not individuals want to receive them [3, 4]. A distinct but equally important question is whether or not patients or research participants should be able to access to their “raw” (uninterpreted) genomic sequence data [5, 6].

A task team of the GA4GH on individual access was established to explore how genomic data generated in both clinical and health research contexts can be more readily shared with individual patients and participants. Research participants primarily want data that is clinically relevant to them or their families [7, 8]. They also attach intrinsic value to genomic data and expect to be able to access data that “belongs to them.” Of 4140 individuals participating in an ongoing international GA4GH survey, 61% would want to be able to access their raw sequence data (with most having the intention to use the data as the basis of further exploration) [9]. Our task team envisages a standard system that allows interested patients and participants to “pull” their genomic data from clinical laboratories or research projects on request. Processes allowing individuals to access *uninterpreted data* are different from policies or processes on the return of individual findings. The latter are premised on the information’s clinical relevance and/or actionability. The right to access uninterpreted data does not undermine the right not to know where it is provided on request. Even so, there are concerns over the accuracy and utility of uninterpreted data, and fears of misuse by individuals or third party services may result in psychological harms or wasted health care resources [10]. Regardless, various research initiatives are opting to provide individual access, most notably the US “All of Us” [11] and UK 100,000 Genomes [12] initiatives, and participatory research projects such as the Personal Genome Project [13]. Drawing on a review of current practice and analysis of the legal right to access personal health data, this paper supplies practical guidance for clinical laboratories or research projects seeking to provide participants access to uninterpreted genomic data. We recognize that it may not always be feasible or appropriate to provide individual access, especially in some (e.g., legacy) research contexts. We predict, however, that individual access will become expected or required as genomics becomes more clinically oriented and the public begins to insist on participatory data governance.

### Current practice

The projects providing or planning to provide individual access to uninterpreted genomic data are listed in Table 1 (adapted from [14]). We were only able to identify one such genomic sequencing project outside of the USA. Data types and formats may differ depending on the context, sequencing platform, analysis pipelines, and evolution of common file formats. The examples of genomic data formats currently provided to participants include reduced BAM, VCF, and FASTQ. The usefulness of the data is enhanced where it is accompanied by rich, standard metadata [15]. Genomic sequencing initiatives may also provide individuals access to their associated health data (phenotypic, clinical, environmental). The choice of file format and the choice of when to provide access should be considered from the perspective of both the project and the individual.

**Table 1:** Projects providing individual access to genomic data

Project	Dates	Context	# Genomes Sequenced to date	Platform	Lab Report with Signout	Results returned	Report to Health Record	Raw Data to Participants	Accredited lab	Last updated
Harvard PGP	2005-	Research	352	WGS	No	Y Filtered Variants w/ Lit Annot	No	Yes (variants)	No	Nov 2017
BWH/Harvard MedSeq	2011-	Research	110	WGS	Yes	Monogenic, Common, PGx	Yes	FASTQ	Yes	Nov 2017
Mount Sinai HealthSeq	2012–2015	Research	40	WGS	No	Monogenic, Common, PGx	No	BAM, VCF	No	paper
Mayo “10 scientists”	2012–2014	Research	10	WES	No	Monogenic	No	Yes	No	paper
Institute for Systems Biology (ISB) Pioneer 100	2014	Research	108	WGS	No	Monogenic, Common, PGx	No	BAM, VCF	No	paper
BWH/BCH/Harvard BabySeq Project	2015–	Research	160	WGS	Yes	Monogenic, PGx	Yes	FASTQ	Yes	Nov 2017
Nevada Institute of Personalized Medicine	2015–	Research	0	WES	Yes	Monogenic, PGx	No	BAM, VCF	No	paper
NYGC Seeq.io	2016-	Research	~500	WGS (ultra low coverage)	No	ancestry, microbiome	No	BAM	No	Feb 2017
NIH All of Us	2017-	Research	0	WGS	?	?	?	?	?	Nov 2017
100,000 Genomes (UK)	2015-	Research	44,633	WGS	Yes	Monogenic, PGx	Yes	Yes^a^	Yes	Jan 2018

Compiled as web site: Bobe, Jason. “sharing-genome-studies,” online: <http://blog.jasonbobe.net/sharing-genome-studies/>

^a^Does not routinely provide access to BAM of VCF files, but participants are allowed to view the files on-site

### A legal right to access?

In many countries, individuals have a legal right to access their personal data held by government bodies and commercial entities [16–18]. A general right to access personal data is included in the EU General Data Protection Regulation (GDPR) (art 15), which comes into force in May 2018 [17]. This internationally recognized right empowers individuals to ascertain what data these entities have about them and how their personal data are used. The right also enables individuals to ensure their data are accurate, up to date, and used in a transparent, fair, and lawful manner. Upon request, individuals must be provided with a copy of their data in a reasonable timeframe, in a useful format, and for a reasonable cost. There is considerable uncertainty and jurisdictional variation over whether or not genetic data is legally considered inherently identifiable. Regardless, genomic data will still fall under broad definitions of personal data used in many jurisdictions (e.g., GDPR art 4(1)), as long as it “relates to” an identifiable individual, which is increasingly the case for linked genomic data in clinical, commercial, and translational research contexts.

Similarly, patients have a legal right to access their health record ([19], art. 19). This ensures transparency in the physician-patient relationship and allows patients to correct inaccurate information (which may be used by third parties such as insurers) or transfer records when changing physicians. Access to health data also empowers patients to take an active role in their health care. Though raw laboratory data are not typically considered part of the health record, this is changing for genomics. In the USA, recent legislative amendments and interpretive guidance extend the right to access under the US federal health privacy law to a broad range of records that may be used to make decisions about individuals, including information generated as part of a laboratory test [20]. For genetic sequencing, this might include “the full gene variant information generated by the test” [21]; for genomic sequencing, the raw sequence data [22]. Genomic sequencing initiatives providing a right to access should indicate this in the consent form, along with the basic information on what is available and how to request access. Consent forms should clearly distinguish between access rights and other communication policies, such as the return of individual findings of clinical relevance [13]. As we discuss below, more detailed guidance can be provided to those individuals requesting access at the point of implementation.

The right of access is generally subject to narrow exceptions: where it would reveal confidential information (about other patients or health professionals), risk serious harm to the individual, or involve disproportionate effort [23]. Providing an individual access to her *own* genomic data would not generally breach professionals’ legal duties of confidentiality to third parties or present serious risks to the individual. An important legal distinction for research contexts is that many countries limit individual access to research data, usually to protect commercial interests and scientific validity [24]. It is often unclear, however, if research exceptions in general access to information provisions were meant to restrict participants from accessing their own data [25]. International and national research ethics guidelines are largely silent about individual access to health data. This is surprising, given that many incorporate other data protection principles [26–28]. Some mention that participants have the right to access their clinical data on demand, unless temporary or permanent non-disclosure is approved by a research ethics committee with reasons ([29], Table 2). Regardless, research exceptions are unlikely to apply as sequencing moves to clinical or hybrid clinical-research contexts. Researchers seeking to provide individuals with access to genomic data may also have to contend with clinical services, clinical laboratory, and/or medical product regulations. The US regulations, for example, require any test results used for clinical decision-making to be done in a certified laboratory [30]. While these restrictions may block the return of clinically relevant individual findings from research laboratories, it is not clear why they would also apply to uninterpreted genomic data.

**Table 2:** Summary of recommendations

Provide access to genomic data in standard formats
1) FASTQ: read-level data
2) BAM: Binary Alignment Map
3) gVCF: Genome Variant Call Format
4) FASTQ: assembled diplotype genome
(Ability to reconstruct genomes: FASTQ ~ = BAM > gVCF > FASTQ)
Provide access upon request unless withholding access is justified (by an Access Office or Research Ethics Committee)
1) Breaching confidentiality of a third party (*could consent from the third party be obtained?*)
2) Imminent and serious harm to the mental or physical health of the individual (*could the harm be mitigated?*)
3) Access compromises a primary objective of a research study (*could access instead be provided at the end of the study?*)
4) Expense compromises the feasibility of a research study (*could participants be asked to cover the costs?*)
Establish appropriate data tracking and security processes
1) Authentication service (e.g., Experian) or in-person account creation
2) Best practices for data security (encryption, user access controls, transfer protocols)
Describe the right to access in the consent form
1) Distinguish from the plan for return of individual findings of clinical relevance
Provide detailed information at the point of access
1) Participant’s right to access uninterpreted data
2) Description of access process
3) Description of risks posed by research-grade data
4) Description of benefits provided by uninterpreted genetic data
5) Description of available genetic counseling services
6) Description of how data will be accessed, stored, and transferred
No warranty and disclaimers
1) Clear articulation that data may not meet clinical standards, and should not be used as a basis for clinical interpretation or decision-making without medical advice and confirmatory testing in an accredited laboratory
2) Clear disclaimers that research sponsors do not offer a warranty of the data accuracy and are not liable for harm caused from using the data
3) Research sponsors should still strive to generate the highest quality data
Funders
1) Incentivize projects to provide participants access
2) Support costs of participant access

In conclusion, it is likely that clinical laboratories have, or will soon have, a legal obligation to provide individuals their raw genomic data upon request. While it is less likely that a legal right applies in research contexts, we propose that projects should still consider providing a default right of participants to access their own individual-level genomic data upon request. Any exceptions to access should be transparently stated, clearly justified, and approved by a research ethics committee or similar body. If access compromises the primary objective of the study, it could be withheld until the objective is achieved. In both research and clinical contexts, data stewards providing individual access should make efforts to ensure data is of high quality and interoperable. Standard use agreements could accompany access explaining that the data is provided “as is,” without implied or express warranties (e.g., that the data is fit for a particular purpose––namely clinical interpretation or decision-making), and disclaiming liability for any harm resulting from the individual’s use of the data.

### Handling ethical and practical concerns

There are many good reasons for researchers to provide access to individual-level uninterpreted data. Empirical studies show that many people believe that their genomic data belongs to them––that they have a right to access, use, and distribute their data as they see fit [31]––even if this contradicts laws or consent forms [32, 33]. Providing access may also build trust and incentivize participation [34]. Moreover, patients are often experts in their condition and may be more motivated to determine the relevance of their health data than researchers focused on discovery [35]. Access will enable curious citizen scientists to explore the myriad meanings of their DNA. Research may even thrive when individuals themselves share data with patient-led registries [36, 37], research projects, or public repositories like openSNP [38, 39] or Open Humans [39]. The usefulness of raw genomic data for the individual will also increase with improvements in data quality and interoperability, expansion of the knowledge base of genotype-phenotype relationships, and the availability of reliable third party services. The more data that is held by individuals, the more portals to connect users to research initiatives [40, 41]; interpretation services to provide ancestry, genealogy, and health or wellness information; and tools to facilitate citizen science and self-driven interpretation [42].

There are, however, concerns third party interpretation services may provide uncertain, potentially inaccurate information of little benefit and may lead to anxiety or unnecessary medical follow-up [43]. To promote responsible use, data stewards could provide individuals who request access information about the limitations of data quality, the limitations of self-directed or third party interpretations, and the importance of secure storage and responsible sharing. In particular, clarity is needed that the data should not be used as a basis for clinical interpretation or decision-making without seeking medical advice and confirmatory testing in an accredited laboratory. User portals could facilitate download and communication, or even direct transfer/donation to trusted storage platforms or research projects. Data stewards should also ensure access processes are privacy protective and secure. They require basic authentication processes (is this actually the participant?); tracking processes (is this actually the participant’s genome?); and a means of re-identifying a genome (how do I break the code?). Researcher confidentiality may be breached if requestors are not properly authenticated, or if data from the wrong genome is returned. Privacy concerns persist after data has been accessed. Individuals may be ill-prepared to keep their own data secure, and third party services may not offer comparable privacy and security protections [44]. Again, research projects could provide individuals with tips on how to safeguard their data. While researchers should do their best to encourage individuals to store and use their data carefully, the ultimate responsibility to do so will rest with the individual.

There are also fears that access may divert resources away from clinical or research activities. Moreover, individuals seeking professional interpretation of their data could be a drain on primary care and genetic services within the health system. This could waste public health system resources and unfairly divert resources to the most proactive, healthy, and educated individuals. Providing access should not, however, necessitate expensive interpretation or counseling, as may be the case for the return of individual results. Costs would be limited to basic tracking, authentication, and communication processes––already common in many laboratory contexts and clinical practices––and download costs.

Currently, many researchers feel they should provide access to individual-level data to patients and participants, but do not have the appropriate resources to do so. To address this problem, research funding bodies could help by providing resources, infrastructure, and incentives. Instead of each project establishing its own system, common data management platforms could be developed to enable individual access (such as those already offered to researchers by direct-to-consumer companies) [45]. Data sharing repositories enabling broad research community access could be modified to enable individual access. Individual access endorsements or badges could recognize laboratory or researcher efforts to share data with interested participants and patients.

## Conclusion

We provide a summary of recommendations for sequencing initiatives providing individual access to uninterpreted genomic data in Table 2. More data and experience is needed to definitively refute paternalist concerns about individuals managing their own genomic data. This will only happen if researchers do what they do best: experiment in a responsible manner to understand how to most appropriately support and enable individual access to genomic data. Here, the variable to tweak is not the data analysis, but the participant communication pipeline. The experiment is off to a promising start.

## Abbreviations

GA4GH: Global Alliance for Genomics and Health
